# Nonspecific Genitourinary Pain Improves after Prostatectomy Using Holmium Laser Enucleation of Prostate in Patients with Benign Prostatic Hyperplasia: A Prospective Study

**DOI:** 10.1371/journal.pone.0098979

**Published:** 2014-06-05

**Authors:** Sung Han Kim, Seung-June Oh

**Affiliations:** 1 Department of Urology, Prostate Cancer Center, National Cancer Center, Goyang, South Korea; 2 Department of Urology, Seoul National University Hospital, Seoul National University College of Medicine, Seoul, South Korea; Oklahoma University Health Sciences Center, United States of America

## Abstract

**Objective:**

To investigate changes in nonspecific genitourinary discomfort or pain (GUDP) before and after holmium laser enucleation of prostate (HoLEP). GUDP associated with lower urinary tract symptoms (LUTS) is a common complaint among benign prostatic hyperplasia (BPH) patients, but very little is known about this clinical entity.

**Methods:**

From February 2010 to August 2011, 100 HoLEP patients with complete clinical data at a single institution were enrolled in the study to analyze the degree of GUDP with a visual analog scale (VAS) from 0 to 10 points at baseline and at 3 and 6 months postoperatively, and to investigate any relationships between GUDP and urodynamics, uroflowmetry, and scores from the International Prostate Symptom Score (IPSS) questionnaire.

**Results:**

Fifty-six patients had LUTS only, while the remaining 44 had both LUTS and GUDP. Pain was located in the suprapubic (42.0%), perineal/penile (33.0%), back (17.0%), and perianal (8.0%) regions. During the post-operative period, at six months, the VAS, IPSS, peak flow rate and post-void residual volume had improved significantly in 44 GUDP patients (p<0.010). GUDP had completely resolved in 40 (90.9%) patients and had decreased in four (9.1%) patients, while seven (12.5%) patients developed GUDP with voiding in the urethral and perineal areas by the third month postoperatively. When compared to patients with complete resolution, those with persistent GUDP were found to have a significantly higher preoperative presence of bladder outlet obstruction (BOO) as an independent risk factor (OR 6.173, 95% CI 1.132–1.323).

**Conclusion:**

Both GUDP and LUTS improved significantly after HoLEP. Patients with significant preoperative BOO tended to have persistent GUDP after surgery.

## Introduction

In recent decades, benign prostatic hyperplasia (BPH) has become increasingly prevalent. Among symptoms other than lower urinary tract symptoms (LUTS) suggestive of BPH, patients often present with complaints of painful urination or discomfort in various nonspecific areas. Several reports have shown that approximately 7.7% to 40% of BPH patients suffer from bothersome genitourinary symptoms, such as discomfort or pain (GUDP), related to voiding [1]–[3].

Genitourinary pain can be manifested in several urological pathologies, such as bladder pain syndrome (BPS), chronic prostatitis/chronic pelvic pain syndrome (CP/CPPS), and overactive bladder (OAB). In clinical practice, clinicians frequently encounter patients with BPH who complain not only of LUTS, but also of pain or discomfort in the suprapubic/lower abdomen and the urethral, scrotal, perineal, or penile areas [2], [4]. We often find that the nature of discomfort in these patients is usually bothersome and most commonly vague and nonspecific, making the pain difficult to classify as BPS, OAB, or CPPS [5], [6].

For those patients with BPH refractory to medical therapy, it has been suggested that surgery, such as transurethral prostactectomy (TURP) and other types of prostatectomy, may improve voiding functions and may also decrease bothersome symptoms of discomfort and pain [4], [7]. However, there is relatively little information regarding GUDP associated with LUTS/BPH. In this study, we sought to describe the characteristics of GUDP in patients with BPH and to document changes in GUDP following holmium laser enucleation of prostate (HoLEP) surgery.

## Materials and Methods

### Ethical Statements

All study protocols were performed according to ethical guidelines of the “World Medical Association Declaration of Helsinki–Ethical Principles for Medical Research Involving Human Subjects”. The prospective study was approved by the institutional review board at Seoul National University Hospital (No. H1301-016-454). All the written informed consents from the patients were obtained.

### Patient Population and Study Design

Clinical records of 144 consecutive refractory BPH patients from February 2010 to October 2011 were reviewed from the institutional prospectively collected BPH database registry. Among the patients in the registry, patients were included for ages over 50 years with complaints of LUTS. The exclusion criteria were as follows: presence of genitourinary cancer, previous genitourinary surgery history, urethral stricture, urinary calculi, urinary tract infection, congenital genitourinary anomaly, primary renal disease, interstitial cystitis/bladder pain syndrome, other neurodegenerative disorders, and those who declined to either respond to the questionnaires or to participate in this study.

All patients underwent the baseline evaluation which included the following components: a general medical history/physical examination for LUTS/BPH including digital rectal examination, International Prostate Symptom Score (IPSS), urinalysis, urine culture in the presence of pyuria, serum creatinine, serum prostate-specific antigen (PSA), transrectal ultrasonography (TRUS), and multichannel video urodynamic study (MMS UD-2000, Medical Measurement System, Ennschede, The Netherlands) to help distinguish obstruction and overactivity of bladder components of the LUTS. In every patient, digital rectal examination was performed to identify suspicious nodules suggestive of malignancy, with TRUS-guided prostate biopsy reserved for those suspected of having prostate cancer. There were no patients with cystoscopic abnormality suggesting interstitial cystitis on preoperative cystoscopy.

All surgical procedures were performed by one urologist (SJO) who experienced more than 100 HoLEP operations at the time of starting this study in the routine manner, as described in a previous publication [8]. The urethral catheter was removed on first or second postoperative day based on intraoperative findings, and patients were discharged after having voided twice with consecutive post-void residual urine volumes less than 100 ml.

All of the patients were asked to rate GUDP with the visual analog scale (VAS) from 0 (no pain) to 10 (worst pain possible) points and to fill out IPSS questionnaire to evaluate LUTS at baseline and at 3 and 6 months post operation. Additionally, patients were instructed to self-assess pain separately from sexual activity, to exclude ejaculation-related discomfort or pain.

### Statistical Analysis

The demographics of patients (LUTS and pain are not demographics) including LUTS (IPSS) and pain (VAS) were analyzed using the Chi-square, paired t, Spearman and Fischer’s exact tests for the correlation between pain and other parameters. Multivariate logistic regression analyses were performed to evaluate significant univariate variables influencing pain. Variables with a 5% level of significance were accepted. All analyses were performed using SPSS for Windows version 18, (SPSS, Inc., Chicago, IL).

## Results

Among a total of 144 patients identified, available clinical data was complete for 100 patients. The mean age of these 100 patients was 68.0 (±5.2) years, with a mean body mass index of 23.7 (±4.2) kg/cm^2^. The mean PSA was 4.3 (±3.9) ng/dL, with total prostate volume of 67.3 (±24.5) mL. Urine cultures were all negative. Additional patient demographics and perioperative variables are summarized in Table 1.

**Table 1:** Baseline demographics.

Parameters (Mean ± SD)	Total (n = 100)	GUDP (n = 44)	No GUDP (n = 56)	P-value*
Age (years)	68.0±5.7	67.1±5.8	69.4±7.0	0.083
Body mass index (kg/m^2^)	23.7±4.2	24.1±3.2	24.0±3.3	0.868
Diabetes (n, %)	10 (10.0)	24 (54.5)	28 (5.0)	0.690
Hypertension (n, %)	30 (30.0)	12 (27.3)	12 (21.4)	0.638
IPSS score				
Voiding symptom	12.4±5.0	11.5±5.7	12.8±4.9	0.295
Storage symptom	8.2±3.7	7.3±3.9	8.9±3.6	0.059
Nocturia	2.6±1.2	2.5±1.2	2.4±1.2	0.275
Quality of Life	4.6±1.1	4.5±1.1	4.7±1.1	0.412
Total symptom	20.8±7.8	18.8±9.1	21.2±7.2	0.068
Uroflowmetry				
Qmax (ml/sec)	9.6±4.7	8.8±4.5	8.1±4.1	0.457
Voided volume (ml)	167.0±113.0	175.3±129.5	160.0±97.7	0.531
Post-void residual (ml)	64.1±91.9	59.2±60.4	77.0±85.2	0.293
PSA (ng/dl)	4.3±3.9	3.9±2.9	3.9±2.5	0.886
Prostate volume (ml)	67.3±24.5	62.1±32.8	75.5±31.6	0.042
TZ volume (ml)	36.8±17.8	34.3±24.6	44.9±25.0	0.047
Urodynamic parameters				
MCC (ml)	361.1±128.9	382.1±125.8	344.7±130.2	0.151
First desire (ml)	185.9±70.9	193.4±89.9	179.7±49.8	0.348
Normal desire (ml)	271.9±99.2	284.2±108.9	261.6±90.1	0.273
Strong desire (ml)	354.6±92.3	345.1±89.4	366.8±106.6	0.306
Compliance (ml/sec)	44.0±23.3	40.6±22.2	46.8±24.0	0.212
BOO index	52.6±28.6	57.8±27.7	48.3±28.8	0.102
PdetQmax (cmH_2_O)	68.7±27.7	73.2±27.5	65.1±27.6	0.147
IDC (n, %)	48 (48.0)	22 (50.0)	17 (30.4)	0.951
BOO in voiding phase (n, %)	94 (94.0)	29 (65.9)	26 (46.4)	0.105
DO in storage phase (n, %)	67 (67.0)	30 (68.2)	37 (66.1)	1.000

IPSS, International Prostate symptom Score; Qmax, peak flow rate; TZ, transitional zone; l cystometic capacity; BMI, body mass index; DM, diabetes; HTN, hypertension; IDC, involuntary detrusor contraction; BOO, bladder outlet obstruction; DO, detrusor overactivity; *, univariate analysis between baseline GUDP and no GUDP.

**Figure 1:**
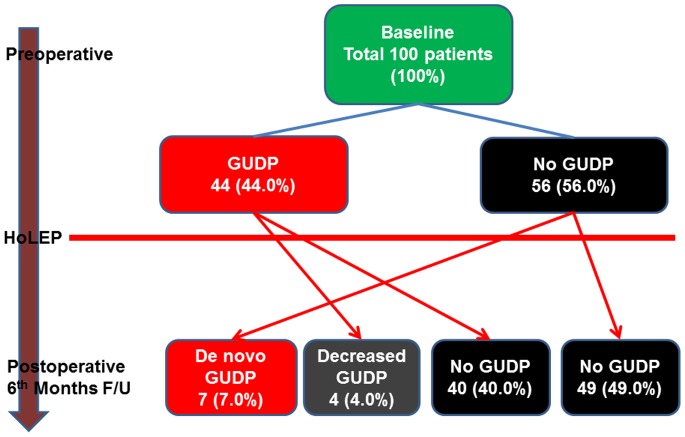
Changes in GUDP from pre to post operation.

Among the 100 patients, 56 (56.0%) had LUTS alone, and the remaining 44 (44.0%) patients had LUTS and GUDP (Figure 1). The location of pain or discomfort included the suprapubic (43.2%), perineal/penile (34.1%), back (15.9%), and perianal (6.8%) areas. At baseline, the visual analog scale (VAS) was 3.2±1.9, and IPSS, peak flow rate (Qmax), and post-void residual urine (PVR) were 18.5±7.6, 10.0±5.3, and 52.8±73.6, respectively. In terms of operative parameters, the mean total operative time was 55.5±29.0 min, with enucleation and morcellation time of 45.1±21.3 min and 11.5±10.2 min, respectively. The mean retrieved weight of prostatic tissue was 24.9±19.6 gm (Table 1).

Postoperatively, at six months, the VAS (0.2±0.8), IPSS (5.1±5.1), Qmax (24.6±11.5 ml/sec) and PVR (17.6±26.5 ml) showed significant improvement in 44 GUDP patients (p<0.010, Table 2). GUDP had completely resolved in 40 (90.9%) patients and had decreased in four (9.1%) patients, while new postoperative GUDP had developed in seven (12.5%) patients at the third month. These seven patients with de novo GUDP complained of pain on voiding in the urethral (n = 4) and perineal (n = 3) areas.

**Table 2:** IPSS and pain change before and after HoLEP (n = 100).

Parameters (mean ±SD)	Preoperative	Postop. 3-mo	Postop. 6-mo	p-value
IPSS				<0.010
Voiding symptom score	12.4±5.0	3.6±4.4	2.4±3.0	
Storage symptom score	8.2±3.7	4.6±2.9	3.4±2.4	
Nocturia score	2.6±1.2	1.6±0.9	1.4±0.9	
Quality of life score	4.6±1.1	1.7±1.5	1.3±1.2	
Uroflowmetry				<0.001
Peak flow rate (ml/sec)	9.6±4.7	18.6±5.7	24.6±11.5	
Voided volume (ml)	166.9±113.0	190.9±118.2	232.6±124.9	
Post-void residual (ml)	52.8±73.6	40.1±35.4	17.6±26.5	
Group (n, %)				
GUDP	44 (44.0)	20 (20.0)	4 (4.0)	<0.001
No GUDP	56 (56.0)	63 (63.0)	89 (89.0)	<0.001
De novo GUDP	NA	7 (7.0)	7 (7.0	NA
VAS score (mean ± SD)	3.2±1.9	0.6±1.1	0.2±0.8	<0.010
Mild pain (1–3) (n, %)	21 (47.7)	23 (85.2)	11 (25.0)	
Moderate pain (4–6) (n, %)	21 (47.7)	4 (14.8)	0	
Severe pain (7–10) (,n, %)	2 (4.6)	0	0	
Total pain sites* (n, %)	44 (100)	27 (100)	11 (100)	<0.010
Bladder, suprapubic area	19 (43.2)	10 (37.0)	4 (36.4)	
Back pain	7 (15.9)	5 (18.5)	0	
Perineum,urethra,distal penis	15 (34.1)	10 (37.0)	7 (63.6)	
Anus	3 (6.8)	2 (7.5)	0	

IPSS, International Prostatic Symptom Score; GUDP, genitourinary discomfort or pain; NA, not available;

*, multiple sites included,

Most cases of GUDP were resolved in patients with severe or moderate pain (>5 on VAS) after HoLEP. The four (9.1%) patients with persistent postoperative GUDP complained of discomfort or pain in either the urethral or perineal areas. Among the 56 patients without preoperative GUDP, seven (12.5%) patients developed new mild postoperative pain (less than three on VAS), and these patients associated their pain with voiding in the urethral and perineal areas.

When compared to patients without preoperative GUDP, those with preoperative GUDP did not have significantly different clinical parameters, with the exception of smaller volumes of prostate and of transitional zones (GUDP group 62.1/34.3 ml ±32.7/24.6 ml vs. no GUDP group 75.5/44.9 ml ±31.6/25.0 ml). However, neither of these parameters was significant in multivariate analysis (p>0.40, data not shown Table).

When compared with patients with complete resolution of GUDP (n = 40), those with persistent GUDP (n = 4) had significantly lower PSA levels, larger maximal bladder capacity, larger volume for bladder sensation (strong desire), higher bladder compliance, and higher prevalence of bladder outlet obstruction (BOO) at baseline (p<0.05, Table 3). Multivariate analysis revealed that preoperative presence of BOO was the only significant risk factor for persistent GUDP following HoLEP (OR 6.173, CI 1.132–1.323, Table 3).

**Table 3:** Significant comparable variables between postoperative persistant GUDP and disappeared GUDP groups among preoperative GUDP patients.

	Disappeared GUDP (n = 40)	Persistent GUDP (n = 4)	Univariate	Multivariate
PSA (ng/dl)	4.0±2.9	2.3±1.1	0.005	0.239
MCC (ml)	375.8±123.5	474.9±146.3	0.033	0.835
Strong sensation (ml)	358.8±99.6	441.8±120.3	0.038	0.839
BOO (n,%)	23 (57.5%)	3 (75%)	0.049	0.040, (OR 6.173, CI 1.132–1.323^++^)
Compliance (ml/sec)	40.1±23.0	58.5±34.4	0.050	0.757

MCC, maximal cystometric capacity; BOO, bladder outlet obstruction; +, the changed differences subtracted IPSS scores from preoperative to postoperative at 6-month; ++, odds ratio (OR) and 95% confidence interval (CI); significant p-values<0.05.

## Discussion

The nature of GUDP in this study population is uncertain. Patients with LUTS/BPH were reported to experience symptoms of nonspecific, vague pelvic discomfort or pain. In some patients, symptoms are transiently associated with micturition, while in others, symptoms are persistent. Because these symptoms are not well characterized, the same set of symptoms can be misdiagnosed as either BPS or CP/CPPS. The nature of pain in patients with BPS is characterized by chronic, recurrent extreme pelvic pain or pressure accompanied with LUTS, which is related to the degree of bladder filling [9]. CP/CPPS is one of four categories of prostatitis characterized by chronic, recurrent pain in the pelvic region in the absence of pathogenic bacteria [9]. A small group of patients with OAB not only present with LUTS, but also with urethral and/or bladder pain^10^. Pain or discomfort among patients with either BPS or CP/CPPS was reported at a mean baseline VAS of greater than 5 [11]–[14]. However, patients with LUTS/BPH plus GUDP in this study had a mean baseline VAS pain score of less than 5. In this regard, detailed pain characteristics of GUDP in our LUTS/BPH patients differ from those of BPS and CP/CPPS. Although the symptoms of pain described by the patients in this study were not typical of CP/CPPS, we cannot completely exclude the possibility that a smaller proportion of these complaints were related to CP/CPPS. The exact pathophysiological mechanism of GUDP should be further investigated in future studies.

LUTS-related pelvic pain and discomfort might be caused by BPH, and surgery might help to reduce pain and discomfort. A few reports have shown that surgery not only improved the LUTS, but also improved pain at nonspecific areas related to the bladder and urethra [7], [15]. In our study, GUDP, when concomitant with LUTS, was relieved by prostatectomy in the majority of patients postoperatively by six months (Table 2). Among the postoperative remnant, including 11 (11.0%) mild GUDP patients (less than 3 scored on VAS for pain), four (4.0%) persistent GUDP patients, and seven (7.0%) de novo GUDP patients, most of the patients with GUDP benefitted from HoLEP in this study. It is worthwhile to note that Kaplan et al. also showed similar findings. In their series, the subjects who were categorized as having chronic nonbacterial prostatitis had, in fact, BOO since transurethral incision of the bladder neck was proved to be an effective treatment [16].

The baseline demographics of enrolled patients demonstrated that GUDP was less severe relative to either BPS or CPPS (baseline VAS 3.2±1.6). Most of the preoperative GUDP scale (95.4%) was of the moderate level (<7 on VAS), and HoLEP reduced GUDP to a mild level (89.0%, Table 2). In those 11 patients with either persistent and de novo GUDP, symptoms may have resolved with a longer follow-up observation. This meant that the characteristics of GUDP in the BPH patients was at a mild to moderate level of painful discomfort relating to LUTS and voiding due to BOO, which differed from that of BPS or CPPS. The GUDP might easily be controllable through surgical procedures.

IPSS scores, uroflowmetry, and VAS were improved postoperatively in an inter-related manner, as reported in a similar study [17] (Table 2). All of the parameters of IPSS, UFM, and VAS improved the subjective symptom and objective voiding evaluations following HoLEP. Improvements in these three parameters suggested that they could be interrelated by the degree to which HoLEP relieves BOO by improving voiding and LUTS.

In spite of the small number of patients to evaluate the relationship between IPSS and urodynamic findings, the first finding of a better response in voiding symptoms in IPSS might be explained by the relationship between BOO and GUDP, which was proven as an independent risk factor found among other significant variables (PSA, maximal bladder capacity, strong desire, bladder compliance, and IPSS scores, p<0.05, Table 3). In a comparison between the postoperative no-GUDP and GUDP groups, higher voiding symptom scores of IPSS in the no-GUDP group were associated with improvements to BOO [18], [19]. The second finding of the worst improvement in nocturia symptom in IPSS might be due to the fact that the nocturia was only partially and indirectly associated with the BOO, but mostly with multi-factors such as hormones, lifestyle, and other physiologic rhythms controlled by the brain [20]–[23]. Our results demonstrated that BOO was a significant and independent risk factor for persistent GUDP following HoLEP. This finding correlated with those of previous studies in which BOO had been identified as an important factor for successful outcomes for GUDP and LUTS following HoLEP.

We would like to describe cases in which patients were diagnosed with small prostates, GUDP, and BOO upon urodynamic findings. Cystourethroscopy revealed that these patients had narrow, high-riding bladder necks, suggesting primary bladder neck obstructions as a cause. In our study, two patients had relatively small prostate volumes (23 and 24 gm) such that the removed prostatic volumes were less than 10 gm. These patients reported satisfactory improvement in LUTS and complete resolution of GUDP following HoLEP.

Among the various manifestations of GUDP included in this study, the back pain of seven (15.9%) patients was the only pain or discomfort arising from outside of the pelvic area that was relieved following HoLEP. Those patients did not have any symptom or signs of pyelonephritis or urinary stones at baseline evaluation before surgery. The seven patients with back pain experienced complete relief from GUDP symptoms in the back or flank areas after relieving the BOO with HoLEP. The exact pathophysiological mechanisms associated with both BOO and back pain are still unknown. However, we propose that any abnormal conditions of the prostate can cause diffuse dull pain or discomfort in the back area, which is commonly observed in chronic nonbacterial prostatitis.

This study investigated the relationship between GUDP and LUTS using VAS to assess pain and IPSS questionnaires to demonstrate a close interrelationship among patients with BPH before and after prostatectomy. The study is among the first research efforts to characterize pain before and after HoLEP and to determine the significance of BOO as it relates to GUDP. However, this study has some limitations to consider. First, this was a relatively short-term, follow-up study with small numbers of patients. Therefore, a prospective long-term, follow-up study with a larger number of patients and a randomized control is needed, especially to evaluate persistent or de novo GUDP patients. Second, to further characterize GUDP in relation to LUTS, more specific and thorough evaluations are necessary to assess GUDP in greater detail. Third, the enrolled patients did not receive specific diagnostic evaluations for CP/CPPS, and there was no further acknowledgment of the likely role of CP/CPPS, such as lower urinary tract localization tests in prostate-specific specimens to exclude CP or laboratory studies on expressed prostatic secretions and post-prostatic massage urine. Therefore, future study with thoroughly acknowledged CP patients among BPH patients could be planned in order to differentiate CP patients’ GUDP from other patients following prostatectomy.

## Conclusions

This study demonstrated that both LUTS and GUDP improved significantly following prostatectomy, and that preoperative BOO may be responsible for GUDP.
